# Differentiation between multifocal CNS lymphoma and glioblastoma based on MRI criteria

**DOI:** 10.1007/s12672-024-01266-9

**Published:** 2024-09-01

**Authors:** Sebastian Johannes Müller, Eya Khadhraoui, Hans Henkes, Marielle Ernst, Veit Rohde, Bawarjan Schatlo, Vesna Malinova

**Affiliations:** 1 Institute of Neuroradiology, University Medical Center, Göttingen, Germany; 2 Clinic for Neuroradiology, Katharinen-Hospital Stuttgart, Stuttgart, Germany; 3 Department of Neurosurgery, University Medical Center, Georg-August-University, Robert-Koch-Straße 40, 37075 Göttingen, Germany

**Keywords:** Central nervous system lymphoma, Multifocal glioblastoma, Brain imaging

## Abstract

**Purpose:**

Differentiating between glioblastoma (GB) with multiple foci (mGB) and multifocal central nervous system lymphoma (mCNSL) can be challenging because these cancers share several features at first appearance on magnetic resonance imaging (MRI). The aim of this study was to explore morphological differences in MRI findings for mGB versus mCNSL and to develop an interpretation algorithm with high diagnostic accuracy.

**Methods:**

In this retrospective study, MRI characteristics were compared between 50 patients with mGB and 50 patients with mCNSL treated between 2015 and 2020. The following parameters were evaluated: size, morphology, lesion location and distribution, connections between the lesions on the fluid-attenuated inversion recovery sequence, patterns of contrast enhancement, and apparent diffusion coefficient (ADC) values within the tumor and the surrounding edema, as well as MR perfusion and susceptibility weighted imaging (SWI) whenever available.

**Results:**

A total of 187 mCNSL lesions and 181 mGB lesions were analyzed. The mCNSL lesions demonstrated frequently a solid morphology compared to mGB lesions, which showed more often a cystic, mixed cystic/solid morphology and a cortical infiltration. The mean measured diameter was significantly smaller for mCNSL than mGB lesions (*p* < 0.001). Tumor ADC ratios were significantly smaller in mCNSL than in mGB (0.89 ± 0.36 vs. 1.05 ± 0.35, *p* < 0.001). The ADC ratio of perilesional edema was significantly higher (*p* < 0.001) in mCNSL than in mGB. In SWI / T2*-weighted imaging, tumor-associated susceptibility artifacts were more often found in mCNSL than in mGB (*p* < 0.001).

**Conclusion:**

The lesion size, ADC ratios of the lesions and the adjacent tissue as well as the vascularization of the lesions in the MR-perfusion were found to be significant distinctive patterns of mCNSL and mGB allowing a radiological differentiation of these two entities on initial MRI. A diagnostic algorithm based on these parameters merits a prospective validation.

## Introduction

Glioblastoma (GB) and central nervous system lymphoma (CNSL) are two different entities occurring in the brain that share several radiological characteristics at presentation on magnetic resonance imaging (MRI). In cases with multilocular presentation, radiological differentiation of these two diagnoses can be particularly challenging. A targeted diagnostic algorithm is needed for this important clinical issue, to prevent unnecessary examinations in these patients. Although staging with computed tomography is an integral part of the work-up for lymphoma, this process is not required for patients with GB. Furthermore, although the diagnosis of lymphoma can sometimes be confirmed by cytopathological examination of cerebrospinal fluid, such diagnosis is rarely possible in patients with GB. The treatment concept of patients with GB also substantially differs from the applied concept in patients with CNSL. While the resection of the contrast-affine tumor represents an important pillar of the treatment strategy for GBs, whenever a tumor resection is reasonably achievable, tumor resection is primarily not attempted in case of CNSL. In patients with CNSL a biopsy is usually performed for histological diagnosis confirmation followed by chemotherapy or radio-chemotherapy. Both GBs and CNSLs are accompanied by a cerebral edema requiring treatment with cortisone, which can compromise the histological diagnosis of CNSL, because tumor cells may no longer be detectable after the treatment. Multiple tumor lesions at manifestation are found in 22–35% of patients with GB; this subgroup has a particularly poor prognosis [1–3]. The subgroup of patients with GB with multilocular manifestation includes multifocal (with a visible connection on T2-/fluid-attenuated inversion recovery (FLAIR)-weighted imaging between the foci) and multicentric (without visible connection between the lesions on imaging) GB [4]. Lymphomas can manifest as primary or secondary CNSL (PCNSL and SCNSL, respectively). Whereas SCNSL refers to a cerebral manifestation of systemic lymphoma, PCNSL affects primarily the CNS. One-third of PCNSLs and 52% of SCNSLs have been reported to occur with multiple lesions [5–7]. Except for the much higher annual incidence of GB than PCNSL (3–5 per 100,000 vs. 0.5 per 100,000 people), both entities share a comparable manifestation age and are found predominantly in males. Regarding radiological characteristics, several imaging criteria for solitaire manifestations have been previously reported, such as the existence of necrosis in GB, lower relative cerebral blood volume (rCBV) in GB than PCNSL, a shoulder-like time-signal intensity curve of PCNSL on MRI perfusion [8], and lower apparent diffusion coefficient (ADC) values of tumor lesions on diffusion-weighted imaging (DWI) [9] in PCNSL than GB. However, no such criteria have been established to date multifocal CNSL (mCNSL) and for GB with multiple foci (mGB). The aim of this study was to evaluate imaging criteria for GB and CNSL with multilocular presentation on MRI, to reliably distinguish between these entities, and to create a diagnostic algorithm based on the identified criteria.

## Methods

### Study design

A retrospective single-center observational study was performed. Institutional review board approval was obtained from the Ethics Committee of University Medical Center Göttingen (approval number 43/4/23). Because of the retrospective, observational nature of the study, informed consent was not necessary.

### Study population

Records of patients with histologically confirmed GB or CNSL treated at our institution between January 1, 2015, and December 31, 2020, were retrospectively reviewed. The main inclusion criteria for patient enrollment were available MRI datasets including a contrast-enhanced T1-weighted sequence, a T2-weighted sequence as well as FLAIR, and a DWI sequence with calculated ADC by the manufacturer. Patients with a single tumor lesion or with more than ten tumor lesions were excluded. A database search identified 462 patients with GB and 176 patients with CNSL. Finally, 72 patients with GB and 67 patients with CNSL matched the criteria (multilocular, initial diagnosis, pre-surgical MRI, GB or CNSL according to the WHO 2021 guidelines). After the exclusion of patients with insufficient MRI datasets (owing to severe movement artifacts, low resolution, or excessive slice thickness) and balancing of the groups (61 GB and 55 CNSL, random exclusion of 11 and 5 cases), 50 patients were finally included in each group. All included GBs were IDH wild type and classified as GBs according to the WHO 2021 guidelines [10]. All included CNSL were histologically classified as diffuse large B-cell lymphomas. In three cases an EBV-association was detected, as described in the literature [11].

### Imaging analysis

The imaging analysis included an evaluation of the contrast-enhanced T1-weighted 3D sequence, T2-weighted/FLAIR sequence, DWI sequence with calculation of the ADC, and, when available, MRI perfusion as well as susceptibility-weighted imaging (SWI). The following radiological parameters were assessed by using the contrast-enhanced T1-weighted sequence: (1) size of tumor lesions (mean diameter in all three dimensions); (2) regularity of the lesions’ edges (regular vs. irregular); (3) contrast medium uptake (homogeneous vs. heterogeneous; weak vs. strong); (4) morphology of the tumor lesions (solid, cystic, mixed with cystic and solid parts, diffuse cortical infiltration, diffuse with stripe-shaped contrast enhancement and diffuse with tree-shaped contrast enhancement; extra-axial tumors were separately counted); and (5) distance between tumor lesions (shortest distance to next lesion).

**Fig. 1:**
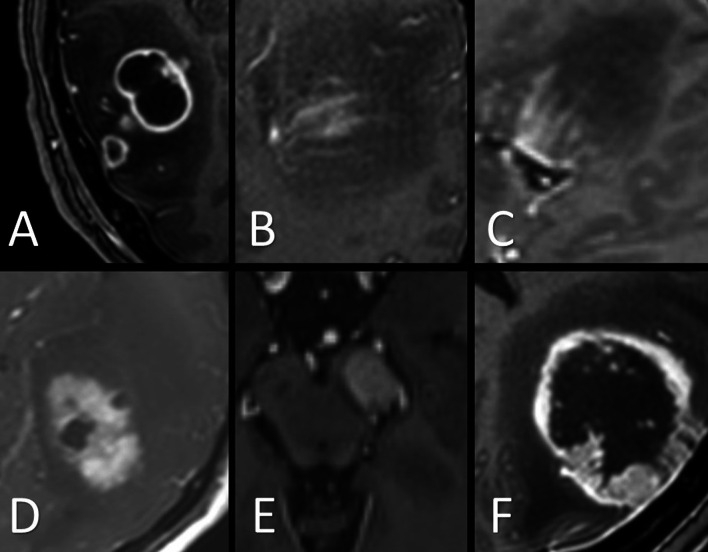
Example of lesion patterns in contrast-enhanced T1-weighted sequences: **A** cystic **B** stripe-like **C** tree-like **D** mixed solid / cystic **E**) solid **F**) solid with central necrosis, counted as mixed solid / cystic. Gb lesions (**A**, **D**, **F**), CNS lymphoma lesions (**B**, **C**, **E**)

Patterns are demonstrated in Fig. 1. The T2-/FLAIR-weighted sequence was used for the evaluation of the following parameters: (1) size of perilesional edema; (2) connections between tumor lesions; and (3) FLAIR: intensity of tumor lesions and perilesional edema (with measurements of contralateral white matter used as reference values [12]). The availability of complete data sets was the main reason for deciding to perform a quantitative evaluation of the T2/FLAIR and not the standard T2. The ADC measurements were also performed within the tumor lesions and perilesional edema zones (with measurements of contralateral white matter used as reference values). To capture the tumor cell infiltration area surrounding the tumor lesion, we determined FLAIR and ADC gradients by measuring these values at three different distances from the tumor lesion: directly adjacent to the tumor at a 5 mm distance; at a moderate distance of 10 mm; and at a long distance of 20 mm (when feasible, depending on the size of the lesion and perilesional edema). For comparison of ADC values in supra- and infratentorial tumor lesions of different field strengths, we used a simple normalization procedure as previously described [13–15]. The imaging analysis was performed by two neuroradiology fellows with more than 3 years’ experience in MRI diagnostics, who were blinded to the clinical information. They independently evaluated the cases and measured the FLAIR and ADC values with GE Centricity™ Universal Viewer (GE Healthcare, 500 W Monroe St, Chicago, IL 60661, United States) software. After identifying MRI characteristics with high discriminatory power, an interpretation algorithm was created based on the identified imaging parameters with the strongest discrimination values according to the Youden’s J index.

### MRI protocol

A complete protocol with MR perfusion following the consensus recommendations [16] for CNS lymphoma was only found in 17 of 50 patients. In 37 of 50 patients with GB, MR perfusion was performed. The small number of correctly carried out protocols can probably be explained by initial misdiagnoses, as multiple GB and lymphomas are rather rare. Since we focus on the morphological characteristics, the lack of MR perfusion was not important for us.

All MR perfusion datasets were MRI DSC T2*, echo-planar “ep2d_perfusion”, either performed on 3 T Magnetom/PrimsaFit or 1.5 T AvantoFit (Siemens Healthineers, Siemensstr. 3, 91301 Forchheim, Germany). The curves were evaluated using syngo.via. MR Perfusion was performed without pre-load, contrast agent (Gadovist, Bayer Vital GmbH, Gebaeude K56, 51366 Leverkusen, Germany) 1 ml per 10 kg of bodyweight, with a flow of 4 ml/s (3 T: TR 1700 ms, TE 28 ms, flip angle 80°, 1.5 T: TE 45 ms, TR 1800 ms, flip angle 80°).

A visual analysis of the perfusion curves was performed. Automated mean curve analyses were not included in the study.

### Statistical analysis

The statistical analyses were performed with the statistical program Statistica, version 13 (TIBCO Software Inc., Palo Alto, California, USA). The significance level was set at *p* < 0.05. The comparison of parameters of mGB and mCNSL was performed with two-sided t-tests. Subgroup analyses for three or more groups were performed via Tukey’s test [17] (Tukey multiple comparisons of means 95% family-wise confidence level) in R Version 4.2.2 (https://r-project.org/). Receiver operating characteristic (ROC) curves and areas under the curve (AUCs) were used for calculating the ADC ratio with the highest diagnostic value in differentiating mGB from mCNSL. The global optimum was determined by maximizing Youden’s J [18]. Correction for multiple comparisons was performed with the Bonferroni method [19].

## Results

### Study population

**Fig. 2:**
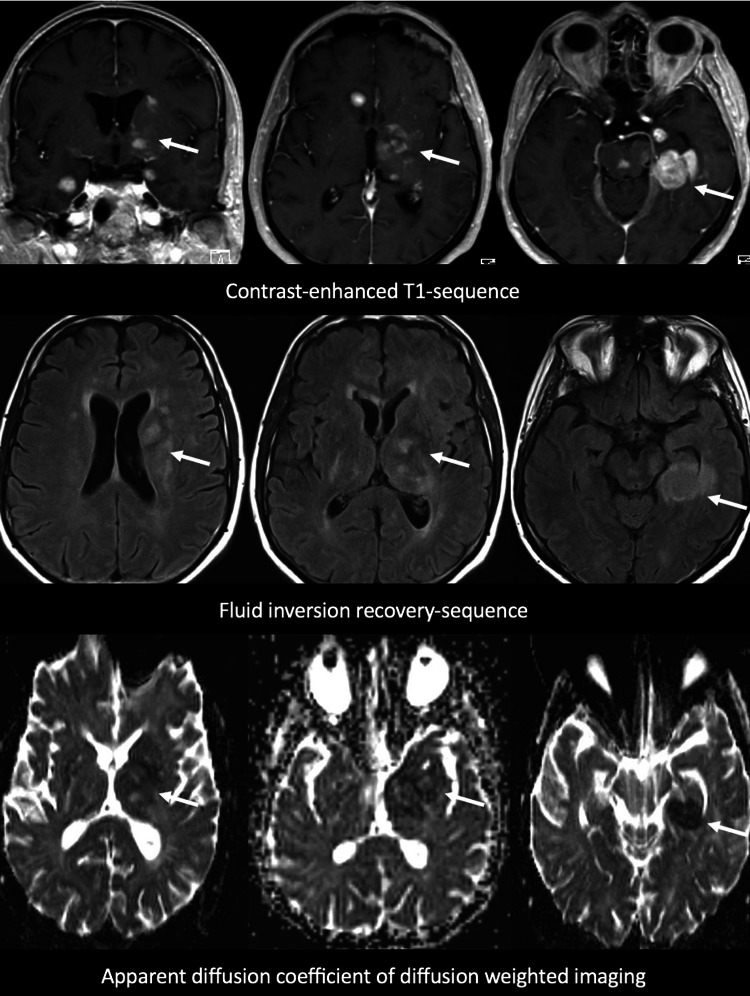
Example of a patient with CNS lymphoma with multiple lesions (mCNSL). Top: contrast-enhanced T1 sequence, showing periventricular tumor growth affecting the basal ganglia, brain stem, and the left hippocampus. Middle: fluid inversion recovery (FLAIR) sequence demonstrating the signal of tumor lesions and perilesional edema. Bottom: diffusion weighted imaging (DWI) with the apparent diffusion coefficient (ADC) indicating a diffusion restriction of the lesions

**Fig. 3:**
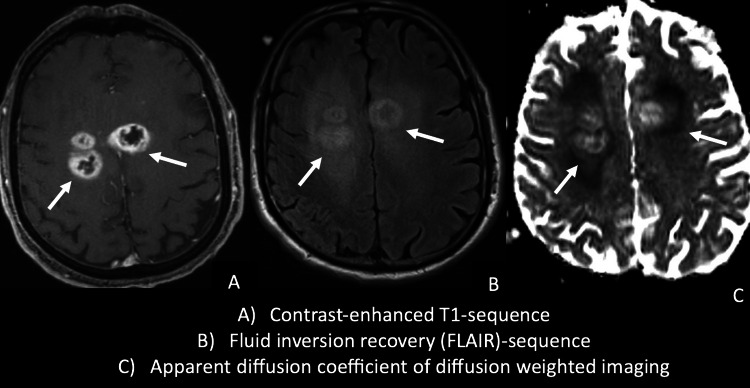
Example of a patient with mGB. **A** Contrast-enhanced T1 sequence showing three separate tumor lesions. **B** Fluid inversion recovery (FLAIR) sequence demonstrating the tumor infiltration zone within the perilesional region. **C** Diffusion weighted imaging (DWI) with the apparent diffusion coefficient (ADC) showing higher ADC values within the tumor lesions but lower ADC values within the perilesional regions

A cohort of 50 patients with mGB with a total of 181 tumor lesions (mean number of tumor lesions per patient, 3.6 ± 1.5; range, 2–7) and a cohort of 50 patients with mCNSL with a total of 187 tumor lesions (mean number of tumor lesions per patient, 3.8 ± 2.4; range, 2–10) were analyzed. The mean age of the mGB subgroup was 65 ± 13 (range, 36–91) years; 68% (34/50) were male, and 32% (16/50) were female. In the subgroup with mCNSL, the mean age was 63 ± 25 (range, 35–81) years; 56% (28/50) were male, and 44% (22/50) were female. PNSCL was diagnosed in 38 patients (76%), and SCNSL was diagnosed in 12 patients (24%). Histologically, malignant diffuse B-cell non-Hodgkin lymphoma was diagnosed in all 50 patients. The baseline characteristics are summarized in Table 1. Typical examples of mCNSL and mGB are shown in Fig. 2 and Fig. 3**.**

**Table 1:** Imaging characteristics

Variables	mGB	mCNSL	*p*-value
Number of tumor lesions	181	187	–
Mean number of tumor lesions per patient ± SD (range)	3.6 ± 1.5 (2–7)	3.8 ± 2.4 (2–10)	0.66
*Location of tumor lesions*			
- Frontal lobe - Parietal lobe - Temporal lobe - Occipital lobe - Diencephalon - Cerebral peduncle - Brain stem - Cerebellum - Corpus callosum - Septum pellucidum - Basal ganglia - Internal capsule - Insula - Ependymal/periventricular - Intraventricular - Leptomeningeal	27% (48/181) 12% (22/181) 22% (40/181) 5% (8/181) 10% (18/181) 0% (0/181) 2% (4/181) 0.5% (1/181) 12% (22/181) 1% (2/181) 3% (6/181) 3% (5/181) 2% (4/181) 0.5% (1/181) 0% (0/181) 0% (0/181)	24% (46/187) 7% (13/187) 12% (23/187) 6% (12/187) 5% (9/187) 4% (7/187) 7% (14/187) 6% (12/187) 12% (24/187) 2% (4/187) 6% (12/187) 0.5% (1/187) 0% (0/187) 7% (14/187) 0.5% (1/187) 1% (2/187)	0.23 < 0.01 < 0.001 0.24 < 0.01 < 0.01 < 0.01 < 0.01 0.47 0.27 0.09 < 0.001 < 0.001 < 0.001 0.34 0.17
*Morphology of tumor lesions*			
- Solid - Cystic and solid - Cystic - Cortical infiltration - Stripe- or tree-shaped	14% (25/181) 14% (25/181) 36% (65/181) 36% (66/181) 0% (0/181)	74% (139/187) 2% (3/187) 6% (11/187) 2% (3/187) 15% (28/187)	< 0.001 < 0.001 < 0.001 < 0.001 < 0.001
Mean size of tumor lesions ± SD (range) in mm	24.8 ± 16.0 (2–83)	18.8 ± 14.3(1–64)	< 0.001
Contrast-enhancement of tumor lesions	77% (139 /181)	88% (165/187)	< 0.001
Mean distance between tumor lesions ± SD (range) in mm	14.0 ± 14.4 (1–73)	19.4 ± 14.7 (1–83)	< 0.001
T2-/FLAIR-connected tumor lesions	63% (114/181)	49% (92/187)	< 0.001
Available MRI-perfusion related to tumor lesions	74% (134/181)	39% (73/187)	< 0.001
Tumor lesions with increased rCBV on MRI-perfusion	63% (85/134)	55% (40/73)	0.16
Available SWI-sequence related to tumor lesions	28% (51/181)	18% (33/187)	< 0.001
Detected hemorrhage within tumor lesions	14% (7/51)	36% (12/33)	< 0.001
Detected increased vascularization	8% (4/51)	52% (17/33)	< 0.001

p-values were calculated via t-test and binomial test

### Radiological characteristics of GB with multiple tumor lesions

Contrast enhancement was found in 77 ± 28% of mGB lesions per patient (range, 0–100%; median, 100%). In 94% (47/50) of mGB cases, the tumor lesions were found to have a supratentorial location, whereas only 6% (3/50) had an infratentorial location. The mean measured diameter on contrast-enhanced T1-weighted sequences of mGB tumor lesions was 24.8 ± 16.0 mm (range, 2–83 mm).

The 181 mGB tumor lesions were morphologically classified as follows: 25 (14%) solid; 25 (14%) mixed, with cystic and solid parts; 65 (36%) cystic; 66 (36%) diffuse cortical infiltration; 0 (0%) stripe- or tree-shaped; and 0 (0%) extra-axial. The mean measured diameter (T2-/FLAIR-hyperintensity) of perilesional edema in patients with mGB was 6.6 ± 8.4 mm (range, 0–40 mm; median, 3 mm). Sixty-three per cent (114 of 181) of mGB lesions were FLAIR-connected to at least one other lesion. The overall mean measured distance between individual tumor lesions in all patients with mGB was 14.0 ± 14.4 mm (range, 1–73 mm; median, 5 mm).

MRI perfusion was available in 74% (37/50) of patients with mGB bearing 134 tumor lesions. An increased rCBV was detected in 85 of 134 (63%) mGB tumor lesions. An SWI sequence was performed in 28% (14/50) of patients with mGB bearing 51 tumor lesions. Hemorrhage was found within 14% (7/51) of assessed tumor lesions, whereas 8% (4/51) of evaluated lesions showed increased vascularization on the SWI sequence. An overview of imaging characteristics in the patient subgroup with mGB is given in Table 1.

#### Subgroup analysis—multicentric versus multifocal GB

In 24% (12/50) of patients with mGB, tumor lesions were found without visible T2-/FLAIR-weighted connections between lesions (“multicentric”), and 76% (38/50) had at least one visible connection between tumor lesions (“multifocal”). To clarify whether there are further morphological differences, we performed this subgroup analysis.

In patients with “multifocal” mGB, the mean distance between the lesions was 9.9 ± 10.4 mm (range, 0–44 mm; median, 2 mm), a value significantly shorter than that in patients with “multicentric” mGB 26.5 ± 17.0 mm (range, 0–73 mm; median, 22 mm; t-test *p* < 0.01). No significant differences among these subgroups were detected in tumor size (“multifocal” mGB, 25.4 ± 16.0 mm; “multicentric” mGB, 22.6 ± 16.2 mm; *p* = 0.14). Perilesional edema was slightly (but not significantly; t-test, *p* = 0.42) more pronounced in “multifocal” mGB tumor lesions (5.9 ± 8.8 mm, range, 0–40 mm; median, 4 mm) than “multicentric” mGB tumor lesions (5.8 ± 6.6 mm, range, 0–19 mm; median, 3 mm). The tumor-edema ratio (lesions without edema were excluded) was higher in the “multicentric” mGB subgroup (5.3 ± 4.9; median, 3.9) than in the “multifocal” mGB subgroup (4.1 ± 3.7; median, 2.6), but the difference was not statistically significant (*p* = 0.17).

### Radiological characteristics of CNSL with multiple tumor lesions

Contrast enhancement was found in 88 ± 33% of mCNSL tumor lesions per patient (range, 0–100%; median, 100%). The mean measured diameter of mCNSL lesions on contrast-enhanced T1 sequences was 18.8 ± 14.3 mm (range, 1–64 mm; median, 16 mm). A visible connection between at least two tumor lesions on FLAIR sequences was found in 49% (92/187) of all lesions. In 36% (18/50) of patients, all tumor lesions were connected on the FLAIR sequence, in 28% (14/50) of patients, the tumor lesions were partly connected, and in another 36% (18/50) of patients, the tumor lesions had no visible connection. The mean measured distance between the mCNSL tumor lesions was 22.8 ± 18.7 mm (range, 1–83 mm; median, 20 mm). The mean measured diameter of perilesional edema was 6.6 ± 8.3 mm (range, 0–44 mm; median, 3 mm). The tumor-edema ratio was 3.9 ± 3.7. The mCNSL tumor lesions were morphologically classified as follows: 74% (139/187) were solid; 2% (3/187) were mixed, with cystic and solid parts; 6% (11/187) were cystic; 2% (3/187) exhibited a diffuse cortical infiltration; 15% (28/187) were stripe- or tree-shaped; and 3% (6/187) were nodular and adjacent to the dura. MRI perfusion was available in 34% (17/50) of patients with mCNSL bearing 73 tumor lesions; an increased rCBV was found in 55% (40/73) of tumor manifestations and in 71% (12/17) of patients with mCNSL. The typical time-signal intensity curve with T1-leakage and high percentage of signal recovery (PSR) was detected in 32% (23/73) of mCNSL tumor lesions (53%; 9/17 patients with mCNSL). An SWI sequence was performed in 20% (10/50) of patients with mCNSL bearing 33 tumor lesions. Hemorrhage within the tumor lesions was detected in 36% (12/33) of tumor lesions and 20% (2/10) of patients with mCNSL with SWI sequence available. Increased vascularization was found in 52% (17/33) of tumor lesions and in 60% (6/10) of patients with mCNSL with SWI sequence available at admission. The imaging characteristics in the patient subgroup with mCNSL are shown in Table 1.

#### Subgroup analysis—primary versus secondary Lymphoma

To clarify whether primary and secondary CNS lymphomas differ morphologically, we performed this subgroup analysis. Multi-parameter analysis (n = 20 parameters) indicated significant differences (mPCNSL vs. mSCNSL, significance level 5%) in the number of FLAIR connections per lesion (0.43 ± 0.50 vs. 0.71 ± 0.46, *p* = 0.0028), distance between lesions (26 ± 19 mm vs. 13 ± 13 mm, *p* < 0.0036) and tumor-edema ratio (3.5 ± 3.2 vs. 5.1 ± 4.8, *p* = 0.047). After correction (Bonferroni, significance level set to *p* = 0.0025), none of these parameters remained significant.

### Differentiation between mGB and mCNSL on initial MRI

The typical time-signal intensity curve with T1-leakage and high PSR was observed only in mCNSL lesions (53% of patients and 33% of tumor lesions), but in none of the mGB tumor lesions (binomial test; *p* < 0.00000001). Hemorrhage (36% vs. 14%, binomial test; *p* = 0.00036) and increased vascularization within the tumor lesions (52% vs. 8%, binomial test; *p* < 0.00000001) were detected significantly more often in mCNSL than mGB tumor lesions. After correction for multiple comparisons (Bonferroni method), the significance level was set to *p* = 0.0025. Whereas mGB lesions were more often marginal contrast enhancing with central necrosis ("cystic" or "cystic and solid" shaped) or presented similarly to low-grade tumor components (“cortical infiltration”), most mCNSL tumor lesions were solid, but 15% showed a pathognomonic “stripe”- or “tree”-like enhancement. The most observed locations of tumor lesions in both entities were similar. Although both entities were associated with a location involving the corpus callosum, mCNSL showed a relative preference for the crus cerebri, cerebellar peduncles, and basal ganglia.

Although the mean measured distance between the foci of mCNSL significantly differed from the mean measured distances in “multifocal” GB (t-test, *p* < 0.001), this was not the case in “multicentric” mGB (t-test, *p* > 0.1). The mean measured diameter of mCNSL tumor lesions was significantly smaller than that in mGB tumor lesions (t-test, *p* < 0.001). The AUC of mean lesion size for differentiation of mGB and mCNSL was 0.62, with an optimized threshold of < 20 mm for detecting mCNSL (Youden's J; sensitivity, 64%; specificity, 56%). No significant differences between mGB and mCNSL were found regarding edema size and the tumor-edema ratio.

The ADC tumor ratios were significantly smaller in mCNSL than in mGB (t-test, *p* < 0.001). The AUC of ADC tumor ratio for differentiation between mGB and mCNSL was 0.63, and the optimized threshold was < 0.87 for detecting mCNSL (Youden's J; sensitivity, 59%; specificity, 64%). A significantly higher ADC ratio of perilesional edema adjacent to the tumor (5 mm) was detected in mCNSL than mGB (t-test, *p* < 0.001). The AUC of the ADC ratio for differentiation of mGB from mCNSL was 0.68, and the optimized threshold was > 1.88 for detecting mCNSL (Youden's J; sensitivity, 62%; specificity, 67%). The more distant ADC ratios (≥ 10 mm) did not significantly differ.

**Fig. 4:**
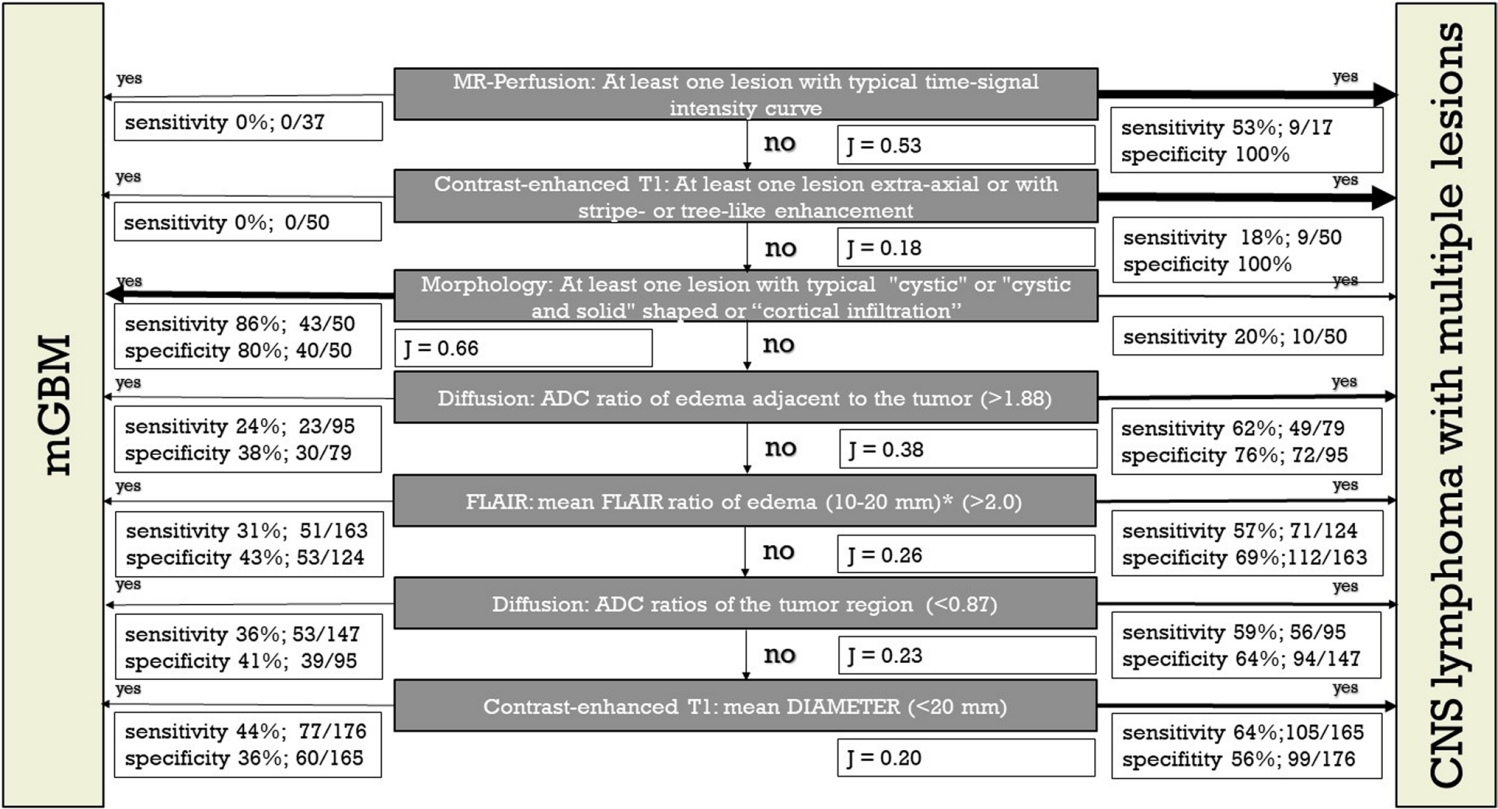
Radiological discrimination flowchart summarizing the distinctive radiological parameters and their discrimination power between tumor entities (*mGB* glioblastoma with multiple foci at presentation, *mCNSL* central nervous system lymphoma with multiple lesions at presentation)

The diagnostic algorithm, as shown in Fig. 4, was built following the rationales: Always consider MR perfusion first. This is followed by properties with a specificity of 100%. Significantly different properties are then arranged in descending order according to Youden’s J.

Table 2 shows the most significant features, evaluated in the decision tree.

**Table 2:** Significant parameters for the differentiation of multiple CNS lymphoma from mGb

Parameter	Accuracy	Sensitivity	Specificity	PPV	NPV
MR perfusion with typical curves*	0.85	0.53	1.00	1.00	0.82
Stripe-like enhancement	0.58	0.16	1.00	1.00	0.55
Morphology: Not one or more lesions (cystic or cystic-solid)	0.84	0.86	0.80	0.81	0.88
Mean ADC-ratio of edema adjacent to the tumor	0.69	0.62	0.76	0.68	0.71
Mean FLAIR ratio of edema	0.64	0.57	0.69	0.58	0.68
Mean ADC ratio of tumor region	0.62	0.59	0.64	0.51	0.71
Mean tumor mean diameter	0.60	0.64	0.56	0.58	0.62

Legend: *—low statistically significance due to the small number of cases (real world values are higher expected); PPV – positive predictive value; NPV – negative predictive value

#### Subgroup analysis

Although the FLAIR ratios of edemas did not significantly differ between mGB and mCNSL, an additional analysis of variance post-hoc test (Tukey’s test) revealed significantly lower FLAIR ratios of perilesional edema in mGB than mPCNSL, but not mSCNSL. The FLAIR values and FLAIR ratios of tumor regions, as well as perilesional edemas, are illustrated in Table 3. The additional subgroup analyses of variance for “multicentric” mGB, “multifocal” mGB, mSCNSL, and mPCNSL are shown in Table 4.

**Table 3:** Subgroup analysis with emphasis on ADC and FLAIR ratios (mean ± standard deviation)

Parameter	mGB (total)	Multifocal mGB	Multicentric mGB	mCNSL (total)	mPCNSL	mSCNSL
Count (lesions)	181	153	28	187	144	43
Infratentorial (lesions)	4	3	1	21	18	3
ADC-ratio (tumor)	1.05 ± 0.35	1.05 ± 0.34	1.06 ± 0.41	0.89 ± 0.36	0.89 ± 0.29	0.90 ± 0.56
ADC-ratio (edema 5 mm)	1.79 ± 0.54	1.77 ± 0.53	1.88 ± 0.55	2.10 ± 1.04	2.13 ± 1.03	1.99 ± 1.06
ADC-ratio (edema 10 mm)	2.07 ± 0.54	2.08 ± 0.52	2.02 ± 0.66	2.23 ± 0.92	2.20 ± 0.93	2.37 ± 0.88
ADC-ratio (edema 20 mm)	2.28 ± 0.50	2.29 ± 0.48	2.24 ± 0.61	2.30 ± 0.77	2.25 ± 0.74	2.65 ± 0.66
FLAIR-ratio (tumor)	1.56 ± 0.29	1.55 ± 0.29	1.64 ± 0.28	1.71 ± 0.48	1.69 ± 0.51	1.77 ± 0.38
FLAIR-ratio (edema 5 mm)	1.80 ± 0.30	1.83 ± 0.28	1.64 ± 0.34	1.92 ± 0.92	1.94 ± 0.93	1.86 ± 0.91
FLAIR-ratio (edema 10 mm)	1.80 ± 0.27	1.83 ± 0.29	1.63 ± 0.08	2.03 ± 0.81	2.03 ± 0.83	2.03 ± 0.72
FLAIR-ratio (edema 20 mm)	1.78 ± 0.27	1.82 ± 0.28	1.64 ± 0.06	2.04 ± 0.69	2.06 ± 0.70	1.97 ± 0.59
Enhancing tumor size (mm)	24.8 ± 16.0	25.4 ± 16.0	22.6 ± 16.2	18.8 ± 14.3	18.3 ± 6.7	20.4 ± 6.0
Edema size (mm)	5.9 ± 8.2	5.9 ± 8.4	5.8 ± 6.6	6.6 ± 8.9	6.7 ± 8.9	6.0 ± 6.4
Mean distance to next enhancing lesion	14.0 ± 14.4	9.9 ± 10.4	26.5 ± 17.0	19.4 ± 14.7	21.6 ± 15.4	12.3 ± 9.2

Legend: mGB – glioblastoma with multiple locations at presentation; mPCNSL/mSCNSL – multiple primary/secondary central nervous system lymphoma; ADC – apparent diffusion coefficient; FLAIR – Fluid Attenuated Inversion Recovery; edemas were measured in 5-, 10- and 20-mm distance (if available) from the contrast enhancing tumor

**Table 4:** Results (p-values) of Tukey’s test (Tukey multiple comparisons of means; 95% family-wise confidence level) of four groups (multifocal mGBM, multicentric mGBM, PNCSL and SCNSL) for T2-FLAIR and ADC ratios

Parameter	ANOVA model Pr(> F)	Multifocal GBM- multicentric GBM	mPCNSL-multicentric GBM	mSCNSL-multicentric GBM	mPCNSL-multifocal GBM	mSCNSL-multifocal GBM	mSCNSL-mPCNSL
ADC-ratio (tumor)	0.012	0.998	0.177	0.446	0.016	0.326	0.999
ADC-ratio (edema 5 mm)	0.006	0.903	0.430	0.950	0.003	0.464	0.776
ADC-ratio (edema 10 mm)	0.452	0.992	0.827	0.605	0.743	0.561	0.880
ADC-ratio (edema 20 mm)	0.632	0.998	0.999	0.678	0.994	0.651	0.572
FLAIR-ratio (tumor)	0.009	0.695	0.948	0.667	0.031	0.041	0.805
FLAIR-ratio (edema 5 mm)	0.026	0.187	0.016	0.263	0.340	0.988	0.870
FLAIR-ratio (edema 10 mm)	0.005	0.446	0.018	0.102	0.042	0.425	0.999
FLAIR-ratio (edema 20 mm)	0.004	0.411	0.013	− +	0.025	− +	− +

Legend: GBM – glioblastoma; mSCNSL/ mPCNSL – multiple primary/secondary central nervous system lymphoma; FLAIR – Fluid Attenuated Inversion Recovery; edemas were measured in 5-, 10- and 20-mm distance (if available) from the contrast enhancing tumor; +—not enough values for analysis

## Discussion

In this comparative study of GB and CNSL with multilocular manifestation, a comprehensive analysis of multiple radiological parameters was performed to identify distinctive imaging features enabling reliable differentiation between these two entities on the initial imaging after presentation. This finding is clinically important because diagnostic work-up and treatment of these tumor entities differ. A heterogeneous presentation of individual tumor lesions on imaging has also been considered to contribute to the difficulty in distinguishing mGB from mCNSL. In this study, we were able to confirm differences between multiple manifestations in DWI and ADC maps that had previously been measured in solitary tumors [9, 20]. We observed slight differences in tumor size and distribution patterns, and significant differences in the ADC ratios of tumor regions and adjacent perilesional edema, as well as in the pattern of contrast agent uptake. A typical time-signal intensity curve on MR perfusion with T1-leakage and high PSR, which is a pathognomonic radiological feature of CNSL, was present in only half the evaluated patients with mCNSL and one-third of the analyzed mCNSL tumor lesions. When present, this radiological feature may aid in differentiating mCNSL from mGB. The findings suggested that MR perfusion is an essential part of MRI for distinguishing between mGB and mCNSL. MRI perfusion, which played only a minor role in our study, will continue to be a leading discriminant in differentiating mGB from mCNSL [8, 21]. Semi-automatic evaluation can already be performed for cases with typical manifestations [22]. In a recently published work by Pons-Escoda et al. the value of dynamic-susceptibility-contrast-perfusion-weighted-imaging (DSC-PWI) was assessed in PCNSL with direct comparison to astrocytoma, meningioma, and metastases. The normalized mean tumor / time-intensity-curves of DCS-PWI showed a good performance for the presurgical identification of PCNSL with an AUC of 0.96 for the direct comparison with glioblastoma, respectively with anaplastic astrocytoma (AUC = 0.83), with metastasis (AUC = 0.95) and meningioma (AUC = 0.93) [23]. These findings are in line with the results of our study with typical time-signal-intensity-curves found only in patients with mCNSL and in none of the patients with mGBs. Automated classification tools were developed which use deep learning algorithm and MR perfusion [24]. Notably, quantitative MRI perfusion (i.e., regional cerebral blood flow measurement) has shown good discrimination between PCNSL and GB [25, 26]. However, problematically, exact quantification requires a sufficiently large lesion, optimal examination conditions, and a fixed perfusion protocol, which are not always available at the time of the initial diagnosis. Differentiation between both cancer types is often simple and can be performed by using machine learning [27] and convolutional networks [28]. Image characteristics of the full spectrum of CNS lymphoma were summarized in a recent review [7], finding an involvement of the basal ganglia, periventricular white matter, midline structures and corpus callosum in up to 45%, as well as a supratentorial emphasis (> 80%).

Although tumor lesions in patients with mCNSL tended to be more often localized within the cerebellum, basal ganglia, or crus cerebri, these locations were not exclusive for mCNSL but were also found in patients mGB. These predominant localizations of lymphoma have been previously reported in a review focusing on the imaging characteristics of this entity [28]. The location of tumor lesions alone can provide a hint but cannot reliably differentiate between the two tumor entities. CNSL lesions often have high cellular density, thus correlating with low ADC values [29]. This finding was also confirmed by our study, which indicated significantly lower ADC ratios within tumor lesions in mCNSL than mGB. In contrast, significantly higher ADC ratios were measured within perilesional edema regions in mCNSL than mGB. Perilesional edema regions of mGB tumor lesions represent the so-called “infiltration zone” encompassing migrating tumor cells, which is expected to be associated with relatively high ADC ratios. This does not apply to mCNSL, thus explaining the lower measured ADC ratios within perilesional regions of mCNSL tumor lesions. A smaller distance between tumor lesions may be indicative of “multifocal” mGB but cannot exclude mCNSL. No significant differences were measured for FLAIR connections and distances. The FLAIR values of “multicentric” mGB significantly differed from those measured in mCNSL. No significant differences between mPCNSL and mSCNSL were found regarding morphology, distribution, size, FLAIR, or ADC properties. The initially identified distinguishing parameters (number of FLAIR connections and distance) did not withstand the correction for multi-parameter analysis.

Previously published studies have reported a diagnostic value of the SWI sequence in patients with suspected lymphomas, in which fewer vessels and a lower intralesional hemorrhage burden are observed in CNS lymphoma [30]. In contrast, we found in our study an increased vascularization and detected more hemorrhage in mCNSL than in mGB. On the one hand, these results may simply be a random shift due to the small number of cases with SWI, on the other hand, it may also be due to the low level of bleeding in mGB. Many morphological studies have been performed by using shape and texture analysis, and have shown good discrimination for typical manifestations [31–33]. In a recently published study using radiomics, several MRI sequences have been evaluated for a reliable differentiation between GB and CNSL. The best discrimination power (AUC 0.97) was found for the combination of an ADC map, T2-/FLAIR sequence, and contrast-enhanced T1-weighted images, whereas SWI and MRI perfusion were not part of the evaluation [34]. These findings are also consistent with the results of our study. New imaging methods, e.g., quantitative mapping, could further facilitate precise differentiation in the future [35, 36].

## Limitations of the study

Many parameters, such as the morphology and regularity of tumor lesions, are determined through subjective evaluation. An automatic assessment based on artificial intelligence, e.g., radiomics, might lead to different results. Because of the retrospective nature of this study, most MRI datasets lacked MRI perfusion and SWI sequence data; consequently, these sequences were considered only as additional parameters. Given the importance of MRI perfusion in this context, complete datasets might have allowed for the development of a more reliable radiological interpretation algorithm. Since the interrater deviations in the initial tests were too large, we decided not to perform a quantitative rCBV evaluation. For the validity of a quantitative measurement of the rCBV or a rCBV ratio [37], the test should ideally be carried out on the same MR scanner. Because of the small number of included patients and the large number of imaging parameters considered, conducting an internal validation of the proposed interpretation algorithm in the study population of the current study was not statistically feasible. An external validation of the algorithm will be mandatory to confirm its utility and reliability in clinical practice.

Furthermore, it should be mentioned that the hyperdense signal of a lymphoma in the computed tomography at initial presentation [7] is also a possible diagnostic criterion, which was not taken into account due to the methodology being limited to MRI.

## Conclusion

We confirmed known differentiating features of solitary GB and CNSL in patients with multiple manifestations on initial MRI, apart from the well-known perfusion criteria. Although several distinctive features were identified, only a few characteristics allowed for reliable differentiation with high accuracy and thus would merit implementation in a radiological interpretation algorithm. Because multifocal tumor manifestations are often difficult to diagnose, this study should facilitate the development of automated decision aids.

## Data Availability

The datasets used and analyzed during the current study are available from the corresponding author on reasonable request.

## Abbreviations

ADC: Apparent diffusion coefficient
AUC: Area under curve
MRI: Magnetic resonance imaging
DWI: Diffusion-weighted imaging
FLAIR: Fluid-attenuated inversion recovery
mCNSL: multiple central nervous system lymphoma
GB: Glioblastoma
mGB: Glioblastoma with multiple foci
PCNSL: Primary CNS lymphomas
SCNSL: Secondary lymphomas
ROC: Receiver operating characteristics
